# Efficacy and long-term effects of intermittent theta burst stimulation on negative symptoms in schizophrenia: a systematic review and meta-analysis

**DOI:** 10.1093/braincomms/fcag027

**Published:** 2026-01-29

**Authors:** Shuyan Tong, Sisi Chen, Jin Chen, Zhu Tong, Wanlong Li, Shasha Liu, Hanqing Shi, Lei Yao, Caiyi Zhang, Xiangrong Zhang

**Affiliations:** Department of Geriatric Psychiatry, The Affiliated Brain Hospital of Nanjing Medical University, Nanjing 210029, Jiangsu, China; Department of Psychiatry, The Affiliated Xuzhou Oriental Hospital of Xuzhou Medical University, Xuzhou 221004, Jiangsu, China; Department of Psychiatry, Xuzhou Medical University, Xuzhou 221004, Jiangsu, China; Department of Psychiatry, The Affiliated Xuzhou Oriental Hospital of Xuzhou Medical University, Xuzhou 221004, Jiangsu, China; Department of Psychiatry, The Affiliated Xuzhou Oriental Hospital of Xuzhou Medical University, Xuzhou 221004, Jiangsu, China; Department of Psychiatry, The Affiliated Xuzhou Oriental Hospital of Xuzhou Medical University, Xuzhou 221004, Jiangsu, China; Department of Psychiatry, The Affiliated Xuzhou Oriental Hospital of Xuzhou Medical University, Xuzhou 221004, Jiangsu, China; Department of Psychiatry, The Affiliated Xuzhou Oriental Hospital of Xuzhou Medical University, Xuzhou 221004, Jiangsu, China; Department of Psychiatry, The Affiliated Xuzhou Oriental Hospital of Xuzhou Medical University, Xuzhou 221004, Jiangsu, China; Department of Psychiatry, The Affiliated Xuzhou Oriental Hospital of Xuzhou Medical University, Xuzhou 221004, Jiangsu, China; Department of Geriatric Psychiatry, The Affiliated Brain Hospital of Nanjing Medical University, Nanjing 210029, Jiangsu, China; Department of Psychiatry, The Affiliated Xuzhou Oriental Hospital of Xuzhou Medical University, Xuzhou 221004, Jiangsu, China

**Keywords:** schizophrenia, negative symptoms, theta burst stimulation, left dorsolateral prefrontal cortex, long-term effects

## Abstract

This study aims to investigate the clinical efficacy and long-term benefits of intermittent theta burst stimulation (iTBS) in alleviating negative symptoms in patients with schizophrenia.

Two authors independently identified eligible studies from the Medical Literature Analysis and Retrieval System Online, Cochrane Library, Web of Science, and Excerpta Medica Database databases up to October 21, 2024. Our meta-analysis was conducted using Review Manager 5.3 and Stata 14 software.

Seventeen randomized controlled trials with a total of 764 participants were included in the meta-analysis. iTBS demonstrated greater efficacy in alleviating negative symptoms immediately after treatment (standardized mean difference = −0.55, 95% confidence interval: −1.00 to −0.10). Improved outcomes were associated with stimulation targeting the left dorsolateral prefrontal cortex, delivery of more than 9900 pulses across over 10 sessions, and use of a stimulation intensity at 80% of the motor threshold. The follow-up results indicated that the iTBS group exhibited greater efficacy than the sham group only at the 6-month mark (standardized mean difference = −0.56, 95% confidence interval: −1.10 to −0.02). iTBS also reduced Positive and Negative Syndrome Scale general and total scores, whereas no significant effect was observed for positive symptoms.

Our meta-analysis suggests that iTBS may alleviate negative symptoms in schizophrenia, with preliminary evidence of long-term benefits observed at the 6-month follow-up.

## Teaser

Tong *et al*. report that intermittent theta burst stimulation targeting the left dorsolateral prefrontal cortex with an optimized protocol alleviates negative symptoms in schizophrenia. Their analysis demonstrates a therapeutic effect evident both post-treatment and at the 6-month follow-up.

## Graphical Abstract

**Graphical Abstract:**
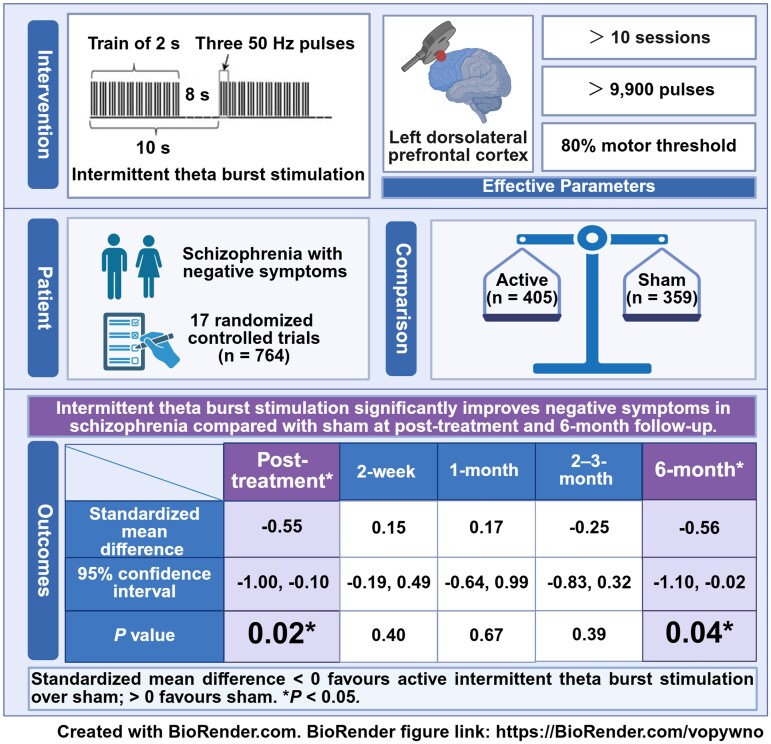

## Introduction

Schizophrenia (SCZ) is a chronic, severe, and debilitating mental disorder characterized by positive, negative, and cognitive symptoms.^1,2^ Negative symptoms, which affect 40–60% of patients, are defined by two core domains: diminished motivation and pleasure (manifesting as avolition, anhedonia, and asociality), and expressive deficits (including blunted affect and alogia).^3,4^ Patients with more pronounced negative symptoms tend to experience poorer functional outcomes, which are closely linked to impairments in occupational, familial and recreational activities, as well as challenges in interpersonal relationships.^5^ Although antipsychotic medications are typically effective in alleviating positive symptoms, treatment options for negative symptoms remain constrained and are often less efficacious.^2^

In recent years, non-invasive brain stimulation (NIBS) has increasingly emerged as a focal point in the research and treatment of negative symptoms of schizophrenia. Repetitive transcranial magnetic stimulation (rTMS), one type of NIBS, demonstrates potential in alleviating these negative symptoms.^6,7^ Theta burst stimulation (TBS) is a patterned form of rTMS that mimics the natural firing patterns of hippocampal neurons^8^ and differs from conventional rTMS protocols in delivery pattern and duration. It is administered in two primary modes: intermittent TBS (iTBS) and continuous TBS (cTBS).^9,10^ The intermittent form, iTBS, delivers 600 pulses in approximately 3 min, offering a substantial time advantage over conventional rTMS.^11^ This efficient protocol has been shown to induce lasting after-effects on cortical excitability,^12^ supporting its investigation as a promising therapeutic strategy for negative symptoms in schizophrenia. However, current research on the efficacy of iTBS in alleviating the negative symptoms of schizophrenia remains inconsistent. This variability may arise from various factors. First, there is no consensus on the optimal cortical area for stimulation. Both the left dorsolateral prefrontal cortex (L-DLPFC)^11,13^ and the cerebellum^14^ have been suggested as potential targets. However, their relative advantages are still debated. Moreover, the discrepancies could also be attributed to variations in the stimulation parameters, such as frequency, intensity, and treatment duration.

Given these uncertainties, we conducted a systematic review and meta-analysis to assess the therapeutic effects of iTBS on the negative symptoms of schizophrenia. To date, the majority of meta-analyses have focused on the efficacy and tolerability of iTBS across various schizophrenia symptoms; however, they have not explored its longitudinal effects.^13-17^ Our meta-analysis provides the first comprehensive evaluation of the long-term therapeutic benefits of iTBS on negative symptoms at different time points.

## Methods

### Protocol and registration

The meta-analysis protocol was registered in the International Prospective Register of Systematic Reviews (PROSPERO) (registration number: CRD420250650645). The study was performed following the Preferred Reporting Items for Systematic Reviews and Meta-Analyses (PRISMA) guidelines.^18^

A comprehensive literature search was conducted before October 21, 2024, across multiple databases, including MEDLINE (PubMed), the Cochrane Library, Web of Science, and EMBASE, to identify all relevant studies for this research.

### Literature search and screening

The following sets of keywords were utilized for the search: (1) ‘schizophrenia’ OR ‘disorder schizophrenic’ OR ‘schizophrenic disorders’ OR ‘schizoaffective’ OR ‘psychosis’; (2)‘randomized controlled trial’ OR RCT OR ‘randomized’ OR ‘controlled’ OR ‘sham-controlled’ OR ‘random’; (3)‘TBS’ OR ‘theta-burst stimulation’ OR ‘iTBS’ OR ‘intermittent theta-burst stimulation.’ Two authors (SY T and SS C) independently conducted the literature search. Subsequently, they meticulously screened the retrieved literature in strict accordance with the inclusion and exclusion criteria.

### Inclusion and exclusion criteria

To ensure transitivity and minimize heterogeneity among the included studies, inclusion criteria were applied^6,11^: (1) randomized controlled trials (RCTs); (2) studies involving the application of iTBS; (3) recruitment of participants diagnosed with schizophrenia or schizoaffective disorder; (4) participants aged ≥18 years; (5) and assessment of negative symptoms in schizophrenia patients.

Exclusion criteria were as follows: (1) studies that were not RCTs; (2) studies that did not assess the severity of negative symptoms; (3) studies that did not recruit participants with schizophrenia; participants aged < 18 years; (4) and in cases of duplicate reporting (i.e. multiple studies based on the same sample), only the study exhibiting the largest sample size was considered for inclusion.

### Data and information extraction

The following data were extracted from the included studies: Demographic information: authors, publication year, study population, number of participants, diagnostic criteria, gender, age, disease duration, and use of antipsychotic medications. Parameters of transcranial magnetic stimulation (TMS): stimulation site, localization method (EEG 10–20 system/MRI-navigated), stimulation protocol, motor threshold intensity, number of pulses, and number of treatment sessions. Scale scores of negative symptoms: the negative subscale of the Positive and Negative Syndrome Scale (PANSS-N),^19^ the Scale for Assessment of Negative Symptoms (SANS),^20^ Brief Negative Symptom Scale (BNSS).^21^ Follow-up outcomes of negative symptoms: post-treatment assessment of negative symptoms. Incidence of adverse reactions: frequency of adverse events (e.g. headache, dizziness).

For data not reported in the articles, the following methods were employed for data collection: (1) Contacting authors: We contacted the corresponding authors via email to request the missing data. (2) Reviewing existing literature: We searched for the data in previously published systematic reviews and meta-analyses. If the required data could not be obtained through these methods, the study was excluded from the analysis.

### Risk of bias assessment

The risk of bias in the included studies was assessed using the Cochrane Risk of Bias Tool version 2.0^22^ (https://methods.cochrane.org/risk-bias-2), covering five domains: (A) randomization process (allocation sequence generation and concealment), (B) deviations from intended interventions (non-protocol deviations), (C) missing outcome data (attrition), (D) outcome measurement (use of validated instruments), and (E) selection of the reported results (consistency with the prespecified protocol and methods).

### Statistical analysis

Meta-analysis was conducted using RevMan 5.3 software. The mean difference (MD) and the standardized mean difference (SMD) were used for continuous variables, while the risk ratio (RR) was used for dichotomous variables. Effect sizes were presented as point estimates and their corresponding 95% confidence intervals (95% CI). Heterogeneity among the included studies was assessed using the *Q*-test (*α* = 0.1) and the *I²* statistic. A fixed-effects model was used if *I*² < 50%, indicating low heterogeneity, and a random-effects model was applied if *I*² ≥ 50%, indicating high heterogeneity. Subgroup analyses were performed based on the following factors: stimulation target site, frequency, number of stimulation trains, and stimulation intensity. Additionally, follow-up effects on negative symptoms were explored to assess the long-term impact of interventions on negative symptoms in schizophrenia patients.

Sensitivity analysis was performed using Stata 14.0 software to assess the stability and reliability of the meta-analysis results. Publication bias was evaluated visually with funnel plots and quantitatively with Egger's linear regression test, with a significance level set at α = 0.05.

## Results

### Study selection and inclusion

**Figure 1:**
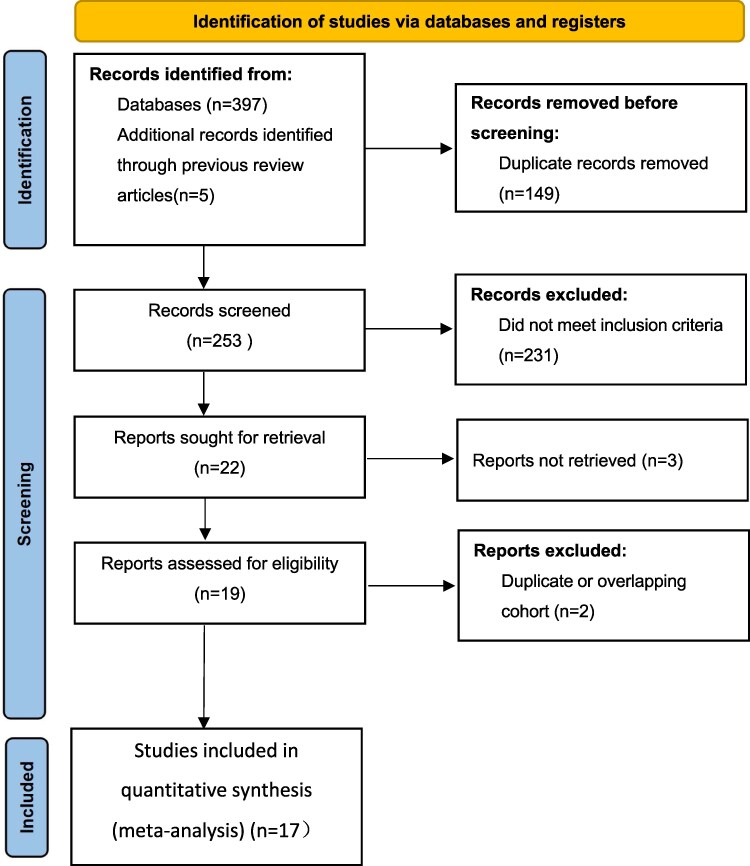
**PRISMA flowchart illustrating the literature screening**. PRISMA, Preferred Reporting Items for Systematic Reviews and Meta-Analyses.

The literature search initially identified 397 articles. An additional five studies were found from previous review articles.^6,11,13,15,16^ After removing duplicates, 253 articles remained for title and abstract screening. Following the review of abstracts, 231 articles were excluded for the following reasons: the study population did not consist solely of patients with schizophrenia, negative symptoms were not assessed, the iTBS protocol was not used, or the study was a meta-analysis or systematic review. As a result, 22 full-text articles were sought for retrieval. Three studies lacked original data despite attempts to contact the authors,^23-25^ and two studies were found to be duplicate publications from the same research team^26,27^ (Wang *et al*., involving the same study population and research parameters). Ultimately, 17 studies met the inclusion criteria (see Fig. 1).

### Study characteristics and clinical demographics

This meta-analysis included 17 RCTs, all involving patients diagnosed with schizophrenia. The diagnostic criteria primarily followed DSM-IV, DSM-V, or ICD-10. The studies were conducted in six countries, with China contributing the most (11 studies). A total of 764 participants were included, with 405 receiving iTBS and 359 receiving sham stimulation. The mean age of the participants was 41.72 years, and the proportion of females was 38.95%. Detailed information is provided in Supplementary Table 1.

All studies employed iTBS as the treatment protocol. The treatment parameters are summarized in Supplementary Table 2. The median treatment duration was 2 weeks, with a median of 10 sessions. The median total number of pulses administered was 9900. These data did not follow a normal distribution. Follow-up assessments were conducted at various time points: three studies reported outcomes 2 weeks post-treatment, four studies at approximately 1 month, three studies at 2–3 months, and three studies at 6 months. Regarding stimulation intensity, nine studies used 80% motor threshold (MT), while seven studies employed 100% MT. The target sites for stimulation varied: ten studies targeted the L-DLPFC,^28-37^ five studies focused on the cerebellar vermis,^38-42^ one study targeted the supplementary motor area,^43^ and one study used the right dorsolateral prefrontal cortex (R-DLPFC) as the stimulation site.^44^ Detailed information can be found in Supplementary Table 2.

Twelve of the included studies reported adverse events. Headache was the most commonly observed adverse effect, followed by dizziness. Other reported adverse events included numbness, tingling sensations, insomnia, and somnolence. Additionally, five studies documented adverse events during the follow-up period (see Supplementary Table 3).

Sixteen included studies used PANSS-N to assess negative symptoms, while one exclusively used the SANS scale. Three studies combined the PANSS-N with the SANS or the BNSS for a more comprehensive evaluation.

### Risk of bias of individual studies

The overall methodological quality varied across studies. Three studies (17.6%) were rated as having a low risk of bias across all domains, while the remaining 14 studies (82.4%) were classified as having ‘some concerns.’ The domains most frequently contributing to elevated risk were the randomization process and the selection of reported results, primarily due to inadequate reporting of allocation concealment and the absence of pre-registered analysis plans. A detailed summary of the risk-of-bias assessment is presented in Table 1.

**Table 1:** Risk of bias summary

Study (Author, Year)	Randomization process	Deviations from intended interventions	Missing outcome data	Measurement of the outcome	Selection of the reported result	Overall Bias
Basavaraju 2021^38^	Low risk	Low risk	Low risk	Low risk	Low risk	Low risk
Bation 2021^28^	Some concerns	Low risk	Low risk	Low risk	Low risk	Some concerns
Brady 2019^39^	Some concerns	Some concerns	Low risk	Low risk	Low risk	Some concerns
Chauhan 2021^40^	Low risk	Low risk	Low risk	Low risk	Low risk	Low risk
Chen 2011^29^	Some concerns	Some concerns	Low risk	Low risk	Low risk	Some concerns
Garg 2016^41^	Some concerns	Some concerns	Some concerns	Low risk	Low risk	Some concerns
Jin 2021^30^	Some concerns	Some concerns	Low risk	Low risk	Some concerns	Some concerns
Jin 2023^31^	Some concerns	Low risk	Low risk	Low risk	Low risk	Some concerns
Kos 2024^44^	Low risk	Low risk	Low risk	Low risk	Low risk	Low risk
Mao 2019^32^	Some concerns	Some concerns	Some concerns	Low risk	Some concerns	Some concerns
Vergallito 2024^33^	Some concerns	Low risk	Low risk	Low risk	Some concerns	Some concerns
Walther 2024^43^	Low risk	Low risk	Some concerns	Low risk	Low risk	Some concerns
Wang 2022^34^	Some concerns	Low risk	Low risk	Low risk	Some concerns	Some concerns
Zhao 2014^35^	Some concerns	Low risk	Low risk	Low risk	Low risk	Some concerns
Zhao 2021^36^	Some concerns	Some concerns	Low risk	Low risk	Some concerns	Some concerns
Zheng 2012^37^	Some concerns	Low risk	Low risk	Low risk	Low risk	Some concerns
Zhu 2021^42^	Some concerns	Low risk	Low risk	Low risk	Low risk	Some concerns

### Individual study results

Among the 17 studies, 12 demonstrated that iTBS was more effective than sham stimulation, while five studies found no significant difference between the two groups.

**Figure 2:**
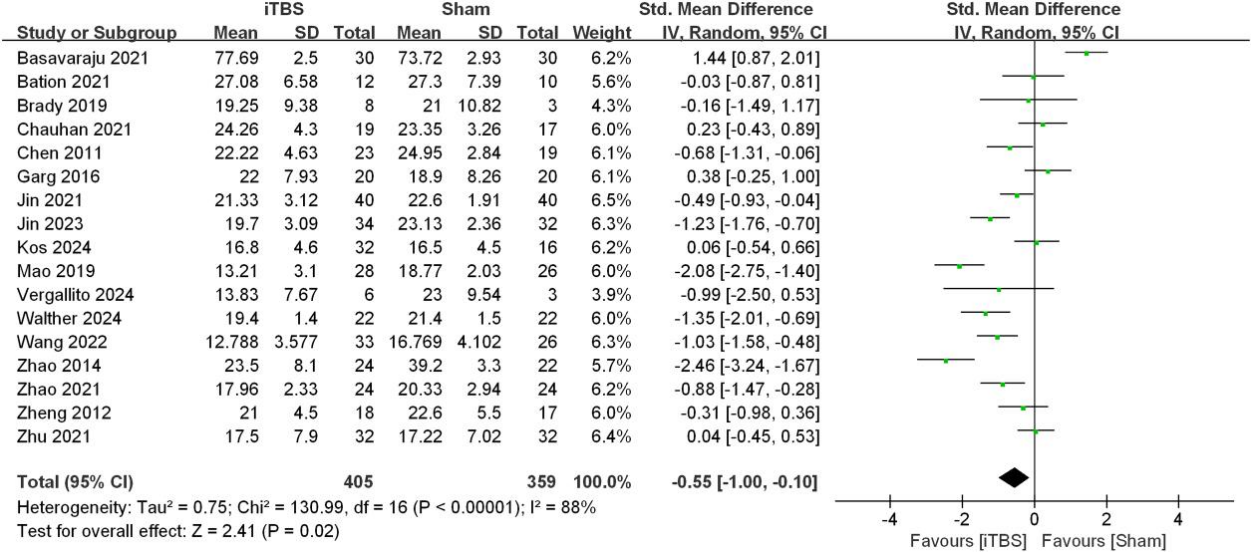
**Forest plot of SMDs for the comparison between iTBS and sham control.** Data are presented for individual studies and pooled using an inverse-variance weighted random-effects model. The total sample sizes are *N* = 405 for the iTBS group and *N* = 359 for the sham group. Each square represents the effect size (SMD) for an individual randomized controlled trial; the horizontal line indicates its 95% confidence interval (CI), and the area of the square is proportional to the study’s inverse-variance weight. The overall effect estimate is represented by a diamond, with 95% CI. Heterogeneity was assessed using the *I²* statistic, and the overall effect was tested with a Z-test. iTBS, intermittent theta burst stimulation; CI, confidence interval; SMD, standardized mean difference; *N*, number of participants; *I²*, heterogeneity statistic.

Heterogeneity: A significant level of heterogeneity was observed between the studies (*I*² = 88%) (see Fig. 2).

### Primary outcomes

The efficacy of iTBS in treating negative symptoms was superior to sham stimulation (SMD = −0.55, 95% CI: −1.00 to −0.10, *P* = 0.02) (see Fig. 2).

Subgroup Analysis (see Table 2): Target Sites (Supplementary Fig. 1): Stimulation of the L-DLPFC demonstrated significantly greater efficacy compared to the cerebellar vermis and other target sites (MD = −4.21, 95% CI: −6.02 to −2.40, *P*  *<* 0.00001). Number of Stimuli (Supplementary Fig. 2): Studies with ≤9900 pulses reported an SMD of 0.2 (95% CI: −0.26 to 0.65, *P* = 0.4), whereas studies with >9900 pulses showed a significantly larger effect (SMD = −1.06, 95% CI: −1.57 to −0.55, *P*  *<* 0.0001). Number of Treatment Sessions (Supplementary Fig. 3): Studies with ≤10 sessions reported an SMD of 0.09 (95% CI: −0.44 to 0.63, *P* = 0.74), whereas studies with >10 sessions demonstrated a significant effect (MD = −4.32, 95% CI: −6.40 to −2.25, *P*  *<* 0.0001). Stimulation Intensity (Supplementary Fig. 4): Studies using 80% MT showed a significant effect (MD = −3.47, 95% CI: −5.99 to −0.96, *P* = 0.007), whereas those using 100% MT did not yield a significant result (SMD = −0.28, 95% CI: −0.99 to 0.42, *P* = 0.43).

**Table 2:** Subgroup analysis results of iTBS treatment for patients with negative symptoms of schizophrenia

		SMD/MD	95% CI	*P* value
**Target site**	L-DLPFC	−4.21	−6.02,−2.40	<0.00001
	Cerebellum	0.44	−0.14,1.02	0.14
	Other sites	−1.23	−3.36, 0.9	0.26
**Number of stimuli**	≤9900 pulses	0.2	−0.26, 0.65	0.4
	>9900 pulses	−1.06	−1.57, −0.55	<0.0001
**Number of sessions**	≤10 sessions	0.09	−0.44, 0.63	0.74
	>10 sessions	−4.32	−6.4, −2.25	<0.0001
**Stimulation intensity**	100% MT	−0.28	−0.99, 0.42	0.43
	80% MT	−3.47	−5.99, −0.96	0.007

Abbreviation: iTBS, intermittent theta burst stimulation; CI: confidence interval; L-DLPFC: left dorsolateral prefrontal cortex; SMD: Standardized mean difference; MD: mean difference.

**Figure 3:**
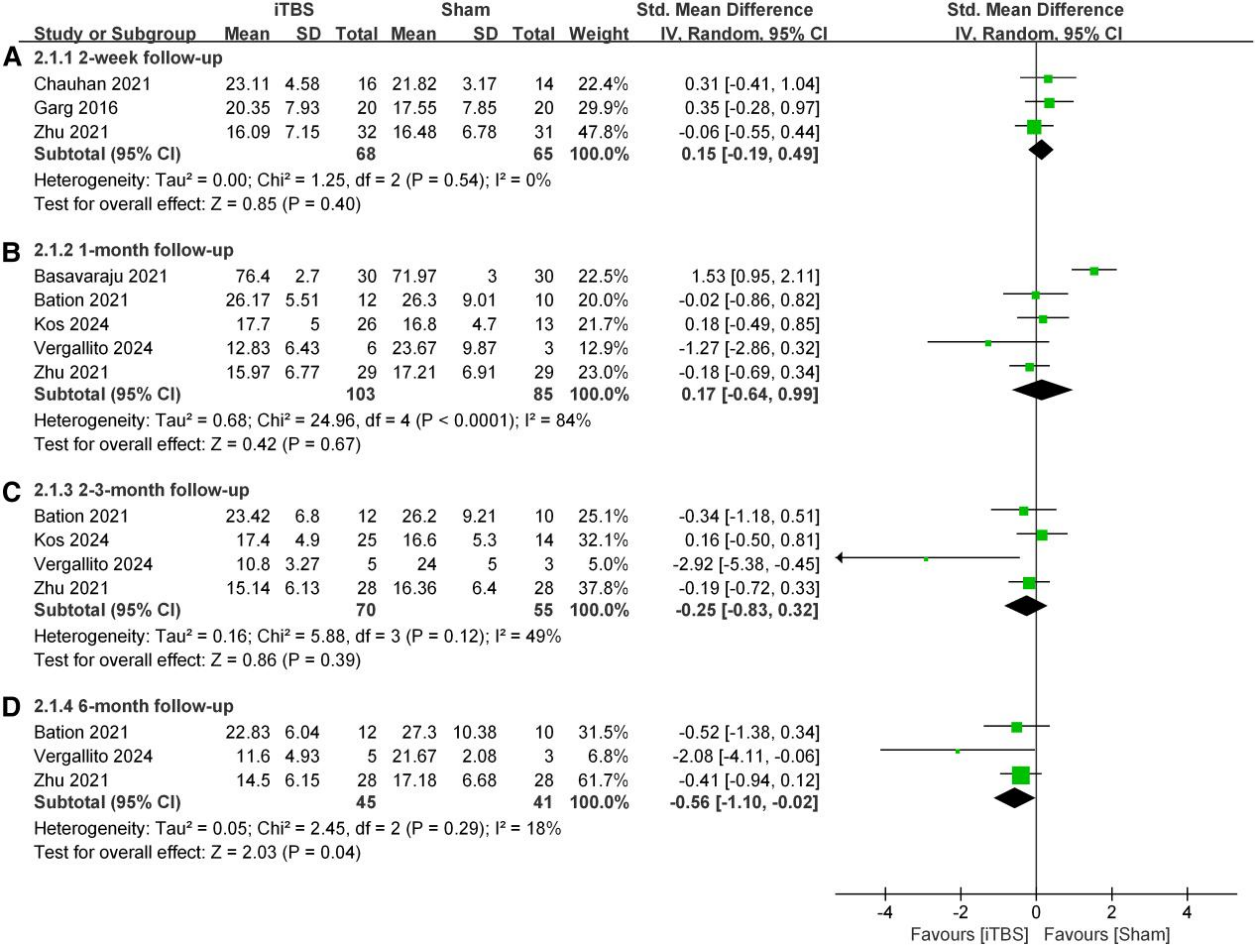
**Forest plot of SMDs for the long-term therapeutic effects of iTBS compared to sham control, stratified by follow-up duration.** Data were pooled for each subgroup using an inverse-variance weighted random-effects model. Each square represents the effect size (SMD) for an individual randomized controlled trial; the horizontal line indicates its 95% confidence interval (CI), and the area of the square is proportional to the study’s inverse-variance weight. Pooled subgroup estimates are represented by diamonds with 95% CIs. Heterogeneity was assessed using the *I²* statistic, and the overall effect was tested with a Z-test. The sample sizes for each follow-up period are: (**A**) 2 weeks (iTBS/sham: *N* = 68/65), (**B**) 1 month (iTBS/sham: *N* = 103/85), (**C**) 2–3 months (iTBS/sham: *N* = 70/55), and (**D**) 6 months (iTBS/sham: *N* = 45/41). iTBS, intermittent theta burst stimulation; CI, confidence interval; SMD, standardized mean difference; *N*, number of participants; *I²*, heterogeneity statistic.

Follow-up Analysis (Fig. 3): We analysed the effects of iTBS on negative symptoms at different follow-up time points: 2 weeks, 1 month, 2–3 months, and 6 months. A significant improvement was observed only at the 6-month follow-up, where the iTBS group outperformed the sham stimulation group (SMD = −0.56, 95% CI: −1.10 to −0.02, *P* = 0.04).

To assess the acceptability of iTBS, a statistical analysis was performed to compare the incidence of the most frequently reported adverse events between the iTBS and sham groups. No significant differences were observed in the incidence of headache (RR = 1.34, 95% CI: 0.83 to 2.14, *P* = 0.23) or dizziness (RR = 1.89, 95% CI: 0.64 to 5.60, *P* = 0.25) between the two groups. Additionally, there was no significant difference in the occurrence of adverse events during the follow-up period between the two groups (see Supplementary Fig. 5).

### Secondary outcomes

In comparison to the sham group, the iTBS group showed significant improvements in the PANSS general symptom score (SMD = −0.39, 95% CI: −0.75 to −0.03, *P* = 0.03) and the PANSS total score (SMD = −0.73, 95% CI: −1.19 to −0.26, *P* = 0.002). However, no significant difference was observed between the two groups in the PANSS positive symptom score (SMD = 0.39, 95% CI: −0.01 to 0.78, *P* = 0.06) (see Supplementary Fig. 6).

### Sensitivity analysis

Notably, the meta-analysis results remained largely unchanged after sequentially excluding individual studies despite some variations in specific indicators. This finding suggests that the overall results are robust and stable (see Supplementary Fig. 7).

### Risk of bias

No significant publication bias was detected based on Egger’s regression test (*P* = 0.853) and funnel plot analysis (see Supplementary Fig. 8).

## Discussion

This systematic review and meta-analysis concluded that iTBS is both practical and safe for treating the negative symptoms of schizophrenia. Treatment protocols targeting the L-DLPFC and involving parameters such as more than 10 sessions, more than 9900 pulses, or stimulation intensities at 80% MT may lead to better outcomes. Notably, this study is the first to evaluate follow-up periods at various stages and investigate the long-term effects of iTBS. The 6-month follow-up results revealed significantly better therapeutic effects in the iTBS group compared to the sham group.

Negative symptoms of schizophrenia (e.g. anhedonia, avolition, asociality) are strongly linked to functional disability^45,46^ and remain insufficiently addressed by current pharmacotherapies.^47^ Intermittent theta burst stimulation, a patterned form of repetitive transcranial magnetic stimulation, has gained attention as a promising neuromodulatory approach.^11^ Its proposed mechanisms of action operate across multiple levels: at the synaptic level, iTBS induces long-term potentiation (LTP)-like plasticity via NMDA receptor-dependent mechanisms and modulation of GABAergic inhibition,^48^ at the network level, it may restore functional connectivity within prefrontal–midbrain^28,45^ and cerebellar–prefrontal circuits^39^; and at the neurochemical level, it modulates striatal dopamine release and regulates glutamatergic balance within the prefrontal cortex.^49,50^ However, the effectiveness of iTBS in treating the negative symptoms of schizophrenia remains inconsistent, with results varying according to factors such as stimulation site, treatment duration, frequency, and intensity.

### iTBS over the L-DLPFC was associated with improved negative symptoms

Current research identifies the primary stimulation targets for treating schizophrenia as the L-DLPFC, the cerebellum, and the medial prefrontal cortex. Our meta-analysis results indicate that stimulation of the L-DLPFC is particularly effective in alleviating negative symptoms, consistent with the findings of previous meta-analyses.^11,13,15^ This may be explained by the neurobiological mechanisms underpinning negative symptoms in schizophrenia. Neuroimaging studies have demonstrated that decreased metabolic activity and inadequate perfusion in the prefrontal cortex, particularly in the dorsolateral prefrontal cortex (DLPFC), are correlated with the negative symptoms of schizophrenia.^51^ The reward-motivation network plays a pivotal role in the pathophysiology of negative symptoms, especially concerning motivational and affective withdrawal.^4^ Key components of this network include the ventral and dorsal striatum, prefrontal cortex (PFC), orbitofrontal cortex (OFC), DLPFC, and the anterior cingulate cortex (ACC).^52,53^ Reduced reward signals within the ventral striatum have been closely associated with apathy. Bation *et al*.^28^ demonstrated that stimulation of the L-DLPFC enhances connectivity with the ventral tegmental area (VTA) and with the right occipital and parietal lobes. This finding suggests that excitatory stimulation of the L-DLPFC may represent a viable therapeutic strategy. Additionally, studies exploring the stimulation of the cerebellar vermis to alleviate negative symptoms of schizophrenia have yielded mixed results. While some studies have reported promising effects, meta-analyses have yet to establish consistent evidence for its efficacy.^14,15^ Bodén *et al*.^25^ investigated the impact of iTBS on the dorsomedial prefrontal cortex (DMPFC) to address anhedonia and blunt affect. The results indicated its effectiveness solely in the depression subgroup, with no observable effects in the schizophrenia subgroup.

### Optimized iTBS parameters for alleviating negative symptoms: higher dose (>10 sessions, >9900 pulses), 80% MT intensity

Common treatment protocols for iTBS typically involve 1–2 daily sessions over a duration of 1–4 weeks. A key question remains whether increasing the number of sessions or extending the treatment duration would yield better therapeutic effects for patients. Based on our meta-analysis, treatment protocols that include more than 10 sessions and exceed 9900 pulses appear to be more effective. The traditional treatment regimen consists of 600 pulses per session daily. In the treatment of depression, derivative modalities such as accelerated iTBS (aiTBS),^54^ prolonged iTBS (piTBS),^55^ and Stanford Accelerated Intelligent Neuromodulation Therapy (SAINT)^56^ have been more extensively studied and implemented. Conversely, in the treatment of schizophrenia, the related clinical research is relatively limited, and the available evidence remains insufficient.

A randomized controlled trial by Jin *et al*.^31^ concluded that accelerated iTBS significantly improves social cognition and negative symptoms in individuals with schizophrenia. Although the precise mechanisms remain to be fully elucidated, the improvements observed following iTBS are theorized to be mediated by changes in metabolic activity, dopamine neurotransmission, and regional cerebral blood flow to the resting brain.^31,57^ Additionally, the improvement in negative symptoms was more pronounced with a 4-week treatment duration compared to 2 weeks. The possible mechanism was that the duration of brain stimulation increased the release of dopamine.

A meta-analysis by Tan *et al*.^13^ concluded that iTBS with a stimulation intensity of 80% MT outperforms 100% MT. Our results align with Tan *et al*.'s conclusion,^13^ but we encountered high heterogeneity, which can be attributed to the lack of standardization in treatment parameters. This difference may be confounded by the distinct stimulation targets commonly associated with each intensity. Studies employing 100% MT frequently targeted the cerebellum, where the increased scalp-to-cortex distance attenuates the induced electric field strength.^13,40^ Consequently, a nominal 100% MT may yield a biologically subthreshold stimulus, thereby reducing efficacy.^13^ In contrast, 80% MT is most commonly applied to the L-DLPFC. One theoretical framework that aligns with this parameter choice is the inverted-U-shaped relationship between stimulation intensity and plasticity^58^: According to this model, excessive stimulation intensity (e.g. 100% MT) may preferentially activate inhibitory processes, whereas moderate levels (approximately 75–80% MT) are thought to more effectively engage facilitatory mechanisms within prefrontal circuits.^58,59^ In summary, iTBS efficacy depends not solely on intensity but on whether the biologically effective dose at the specific target sufficiently engages the intended neuroplastic mechanisms. Thus, these conclusions necessitate further investigation through larger-scale, more robust RCTs.

### iTBS demonstrates long-term therapeutic benefits at the 6-month follow-up

To the best of our knowledge, this meta-analysis represents the most comprehensive synthesis to date of evidence on the long-term efficacy of iTBS in improving negative symptoms of schizophrenia across multiple follow-up intervals. Improvement in negative symptoms was observed at the 6-month follow-up, with no clear benefit at earlier follow-up assessments.

Despite heterogeneity in participant characteristics, the 6-month improvement appears biologically plausible and is consistent with a delayed neuroplasticity mechanism. Bation *et al*.^28^ reported greater iTBS efficacy at 6 months, attributing the delayed response to reduced neuroplastic capacity in older, chronic patients and to the gradual trajectory of functional recovery. Zhu *et al*.^42^ suggested that iTBS may progressively restore prefrontal–cerebellar connectivity through the cerebello–thalamo–cortical pathway, consistent with the differential neuromodulation hypothesis. Comparable long-term effects with other rTMS protocols^60,61^ further reinforce that network-level adaptations require extended time to translate into measurable clinical improvement.

The absence of significant early effects may be explained by several factors. The trait-like and enduring nature of negative symptoms suggests that functional recovery is inherently gradual,^62^ and short follow-up durations may be insufficient to detect latent improvement. For instance, Basavaraju *et al*.^38^ observed enhanced cerebello–prefrontal connectivity at 6 weeks without symptomatic improvement, suggesting that neuroplastic changes may precede clinical benefits. Moreover, Chauhan *et al*.^40^ noted that suboptimal stimulation parameters might have failed to sufficiently engage target regions or induce sustained neuroplasticity. Neuroplastic adaptations are progressive and require sufficient time to consolidate into observable clinical outcomes.^28,42,63^Additionally, although statistically nonsignificant, consistently higher baseline symptom scores in active iTBS groups may have partially masked early effects.

In summary, the six-month benefit likely reflects the intrinsically slow course of neuroplastic reorganization and the persistent nature of negative symptoms, as iTBS-induced synaptic and network remodelling requires time to consolidate into meaningful functional recovery.

### iTBS shows favourable patient tolerability

Our findings suggest that iTBS appears to have a favourable safety profile and is generally well tolerated by patients. The most commonly reported adverse events included headache, dizziness, numbness, and tingling sensations, all of which were transient and resolved spontaneously. Moreover, the incidence of adverse effects and dropout rates during the follow-up period were comparable between the iTBS and sham groups. These findings, which are consistent with those of previous meta-analyses,^15,17^ support the favourable tolerability of iTBS in clinical applications.

### Limitations and outlook

Our meta-analysis has several limitations. Firstly, the heterogeneity is relatively high: the studies included in our analysis exhibit significant heterogeneity (*I*² = 88%), which may be attributed to differences in study design, treatment parameters (such as stimulation frequency, intensity, target areas, etc.), and the characteristics of populations (e.g. age of onset, disease duration, and severity). Secondly, our study does not address the derivative therapeutic applications of iTBS. Current neurostimulation techniques, including aiTBS, piTBS, and the SAINT protocol, remain underexplored in the context of schizophrenia spectrum disorders. Further clinical trials are necessary to investigate the negative symptoms in schizophrenia. A comprehensive evaluation of these methods could yield valuable therapeutic insights and inform evidence-based protocols for managing negative symptoms in schizophrenia. Thirdly, the relatively small sample size (17 RCTs, 764 participants) may limit the stability and generalizability of the findings. Inclusion of studies with ‘some concerns’ in risk of bias may weaken the evidence. Future studies incorporating a larger number of low-risk RCTs are needed to strengthen the reliability and validity of these findings. Fourthly, there are limitations in the study design. All the included studies allowed concurrent use of antipsychotic medication during the study period, which may have impacted the independent assessment of iTBS efficacy. Although our meta-analysis revealed no statistically significant difference in antipsychotic medication use between the iTBS and sham stimulation groups(see Supplementary Fig. 9), potential medication effects cannot be entirely ruled out. Fifthly, there is limited exploration of the treatment mechanisms, and further research needs to investigate areas such as neuroimaging, electroencephalographic activity, neurotransmitters and peripheral biomarkers. This will facilitate the development of individualized TMS treatment protocols. Lastly, there is a lack of long-term outcome data. Although our review is the first to explore the long-term treatment effects at different time points, the limited number of studies in this area means the findings remain relatively conservative. Our findings highlight the need for further research on the long-term effects of iTBS.

## Conclusion

This meta-analysis suggests that iTBS may represent a potentially effective and well-tolerated intervention for negative symptoms in schizophrenia, particularly when administered with specific stimulation parameters, including left dorsolateral prefrontal cortex targeting, more than 10 sessions, delivery of over 9900 pulses, and an intensity at 80% of the motor threshold. Furthermore, iTBS may demonstrate long-term therapeutic effects at the 6-month follow-up. However, further large-scale randomized controlled trials are necessary to verify its efficacy in improving negative symptoms, assess long-term effects, and explore the mechanisms underlying its therapeutic effects.

## Supplementary Material

fcag027_Supplementary_Data

## Data Availability

As this study analysed pooled, de-identified data contributed by multiple research teams across different countries, data availability is structured in tiers. The core data supporting the findings of this study are available within the article and its Supplementary material. Access to the full, study-specific datasets can be obtained upon reasonable request to the corresponding author, who will coordinate with the respective contributing investigators.
