# Supplementary material for: Efficacy and long-term effects of intermittent theta burst stimulation on negative symptoms in schizophrenia: a systematic review and meta-analysis
Source: Brain Commun. 2026 Jan 29;8(1):fcag027. doi: 10.1093/braincomms/fcag027 (PMC12906989; doi:10.1093/braincomms/fcag027)
Supplement: fcag027_Supplementary_Data [file fcag027_supplementary_data.docx]

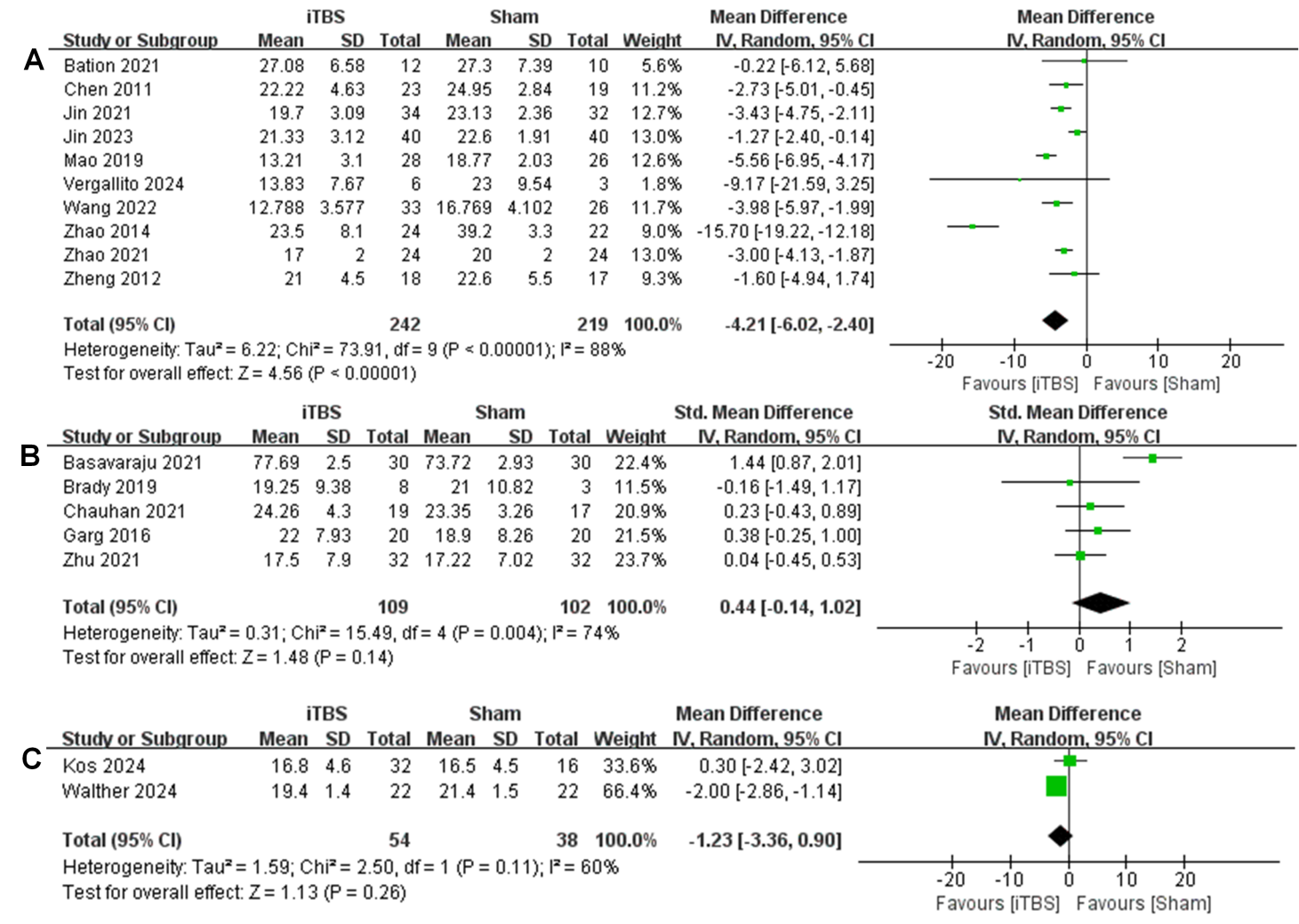


**Supplementary Figure 1: Forest plot of the effects of iTBS on negative symptoms, stratified by stimulation site**. The L-DLPFC and other-site subgroups were analysed using MD, whereas the cerebellar subgroup was analysed using SMD. Each square represents the effect size (MD or SMD, as appropriate) for an individual randomized controlled trial; the horizontal line indicates its 95% confidence interval (CI), and the area of the square is proportional to the study’s inverse-variance weight. Pooled subgroup estimates are represented by diamonds with 95% CIs. Heterogeneity was assessed using the *I²* statistic, and the overall effect was tested with a Z-test. Data were pooled for each subgroup using an inverse-variance weighted random-effects model. The sample sizes for each stimulation site subgroup are: (A) L-DLPFC (iTBS/sham: N = 242/219), (B) cerebellum (iTBS/sham: N = 109/102), and (C) other sites (iTBS/sham: N = 54/38). iTBS, intermittent theta burst stimulation; CI, confidence interval; MD, mean difference; SMD, standardized mean difference; L-DLPFC, left dorsolateral prefrontal cortex; N, number of participants; *I²*, heterogeneity statistic.

**
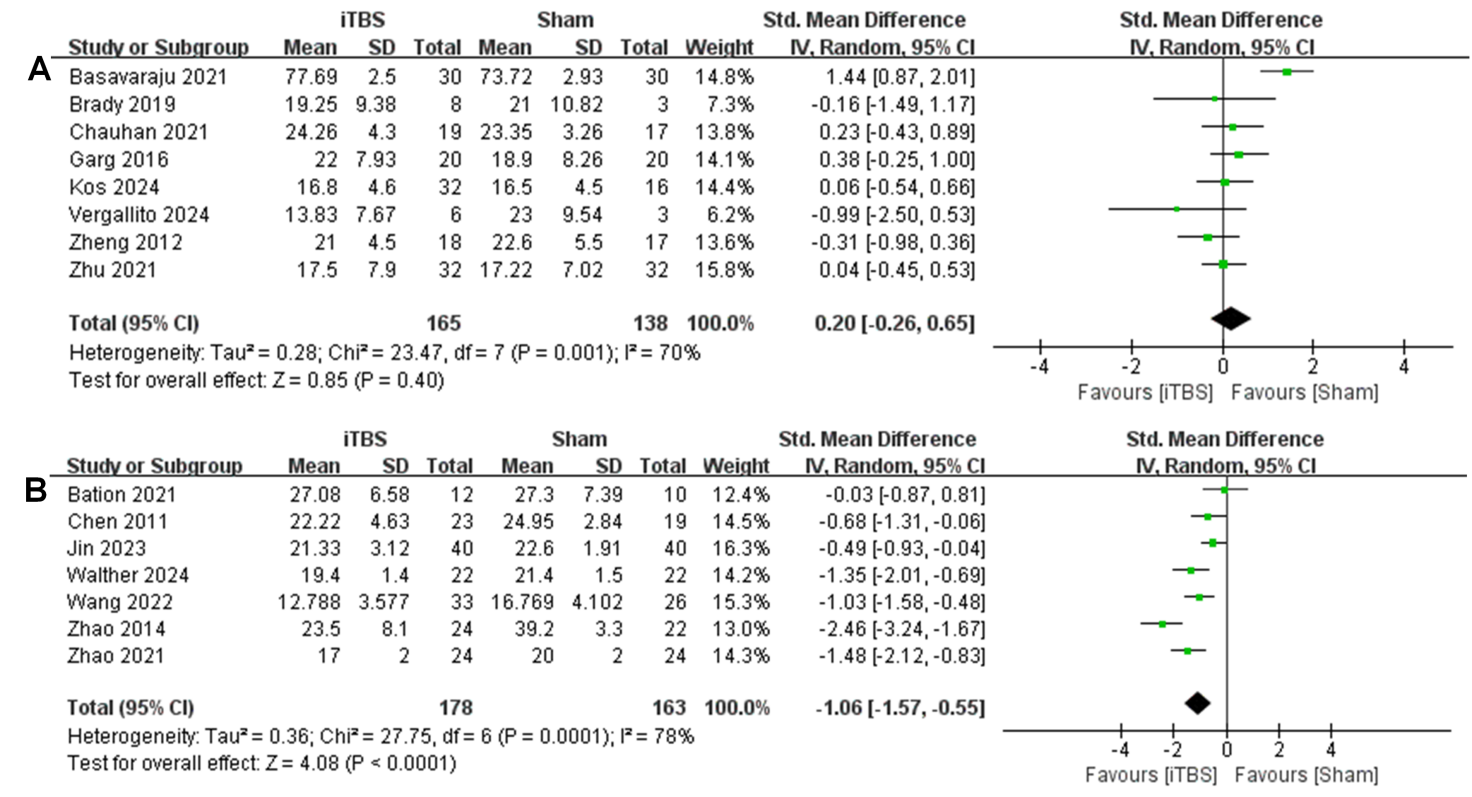
**

**Supplementary Figure 2: **Forest plot of** SMDs for **the effects of iTBS on negative symptoms, stratified by total number of pulses.**** Data were pooled for each subgroup using an inverse-variance weighted random-effects model. Each square represents the effect size (SMD) for an individual randomized controlled trial; the horizontal line indicates its 95% confidence interval (CI), and the area of the square is proportional to the study’s inverse-variance weight. Pooled subgroup estimates are represented by diamonds with 95% CIs. Heterogeneity was assessed using the *I²* statistic, and the overall effect was tested with a Z-test. The sample sizes for each pulse-dose subgroup are as follows: (A) ≤9900 pulses (iTBS/sham: N = 165/138) and (B) >9900 pulses (iTBS/sham: N = 178/163). iTBS, intermittent theta burst stimulation; CI, confidence interval; SMD, standardized mean difference; N, number of participants; *I²*, heterogeneity statistic.

****
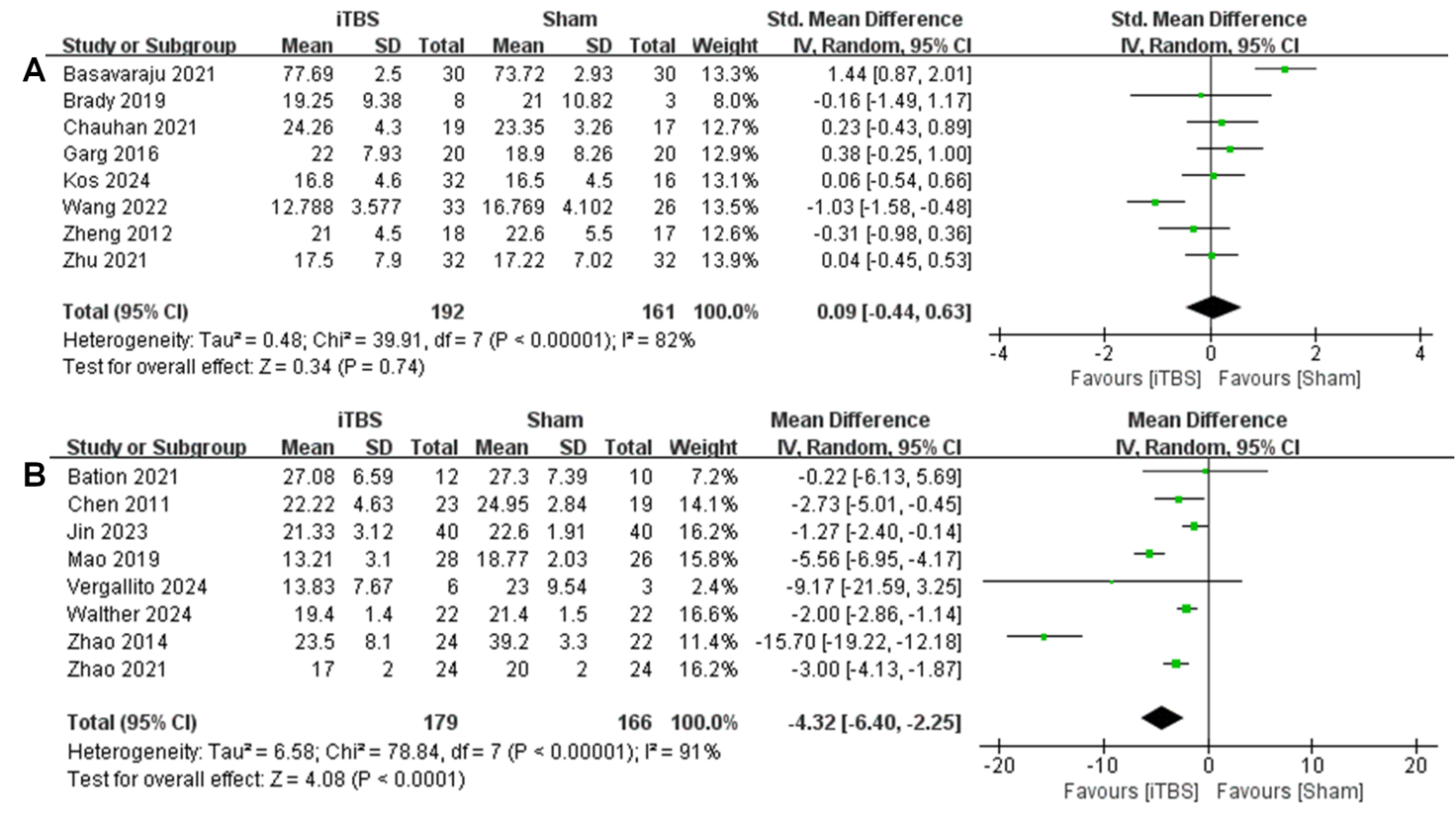
****

**Supplementary Figure 3: Forest plot of the effects of iTBS on negative symptoms, stratified by number of treatment sessions.** The analysis of the >10-session subgroup used MD, while the ≤10-session subgroup used SMD. Data were pooled for each subgroup using an inverse-variance weighted random-effects model. Each square represents the effect size (MD or SMD, as appropriate) for an individual randomized controlled trial; the horizontal line indicates its 95% confidence interval (CI), and the area of the square is proportional to the study’s inverse-variance weight. Pooled subgroup estimates are represented by diamonds with 95% CIs. Heterogeneity was assessed using the *I²* statistic, and the overall effect was tested with a Z-test. The sample sizes (iTBS/sham) for each subgroup are: (A) ≤10 sessions (N = 192/161) and (B) >10 sessions (N = 179/166). iTBS, intermittent theta burst stimulation; CI, confidence interval; MD, mean difference; SMD, standardized mean difference; N, number of participants; *I²*, heterogeneity statistic.


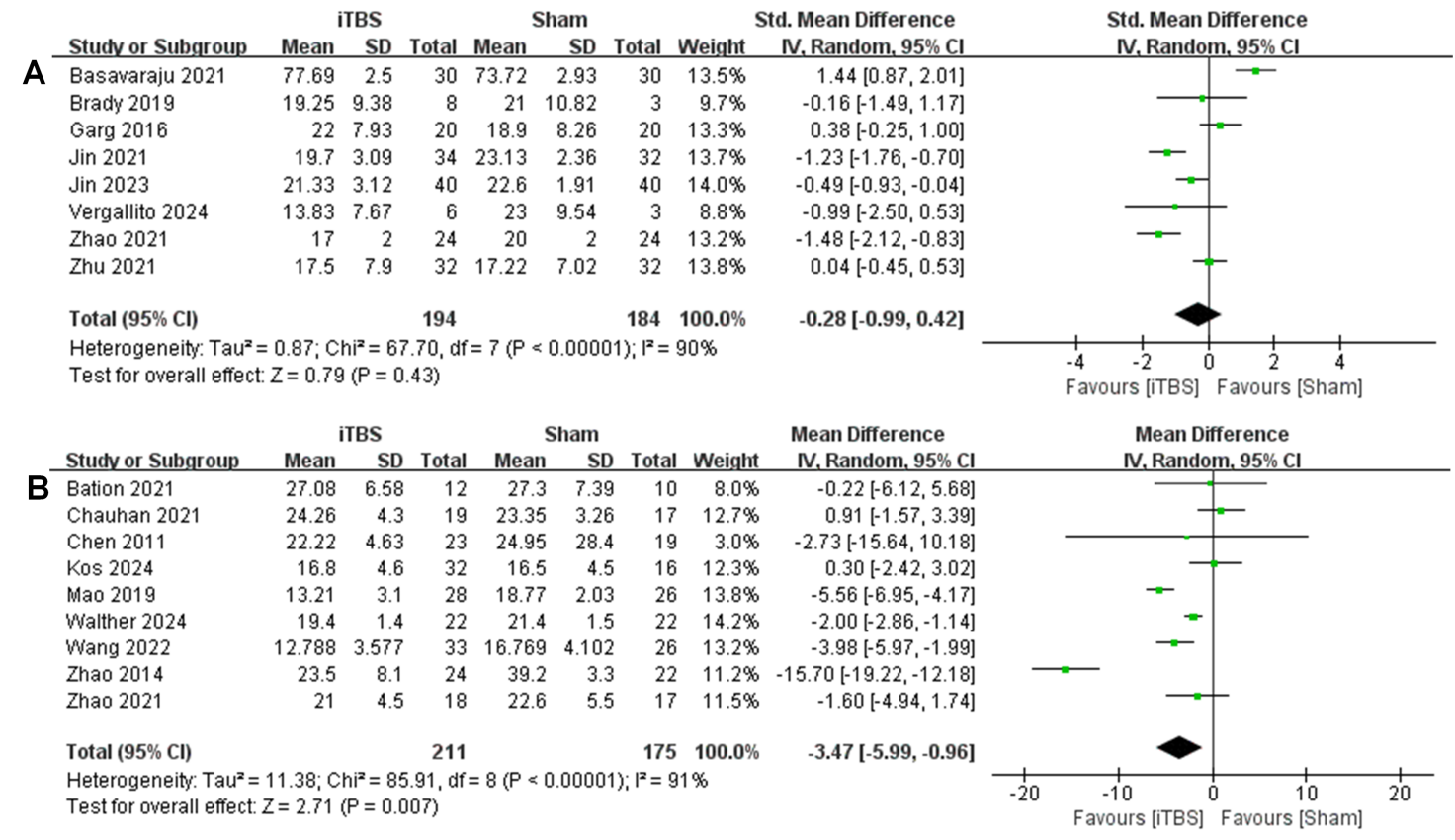


**Supplementary Figure 4: Forest plot of the effects of iTBS on negative symptoms, stratified by the percentage of motor threshold used for stimulation.** The analysis of the 80% MT subgroup used MD, while the 100% MT subgroup used SMD. Each square represents the effect size (MD or SMD, as appropriate) for an individual randomized controlled trial; the horizontal line indicates its 95% confidence interval (CI), and the area of the square is proportional to the study’s inverse-variance weight. Data for each subgroup were pooled using an inverse-variance weighted random-effects model. Pooled subgroup estimates are represented by diamonds with 95% CIs. Heterogeneity was assessed using the *I²* statistic, and the overall effect was tested with a Z-test. The sample sizes (iTBS/sham) for each subgroup are: (A) 100% MT (N = 194/184) and (B) 80% MT (N = 211/175). iTBS, intermittent theta burst stimulation; CI, confidence interval; MD, mean difference; SMD, standardized mean difference; MT, motor threshold; N, number of participants; *I²*, heterogeneity statistic.


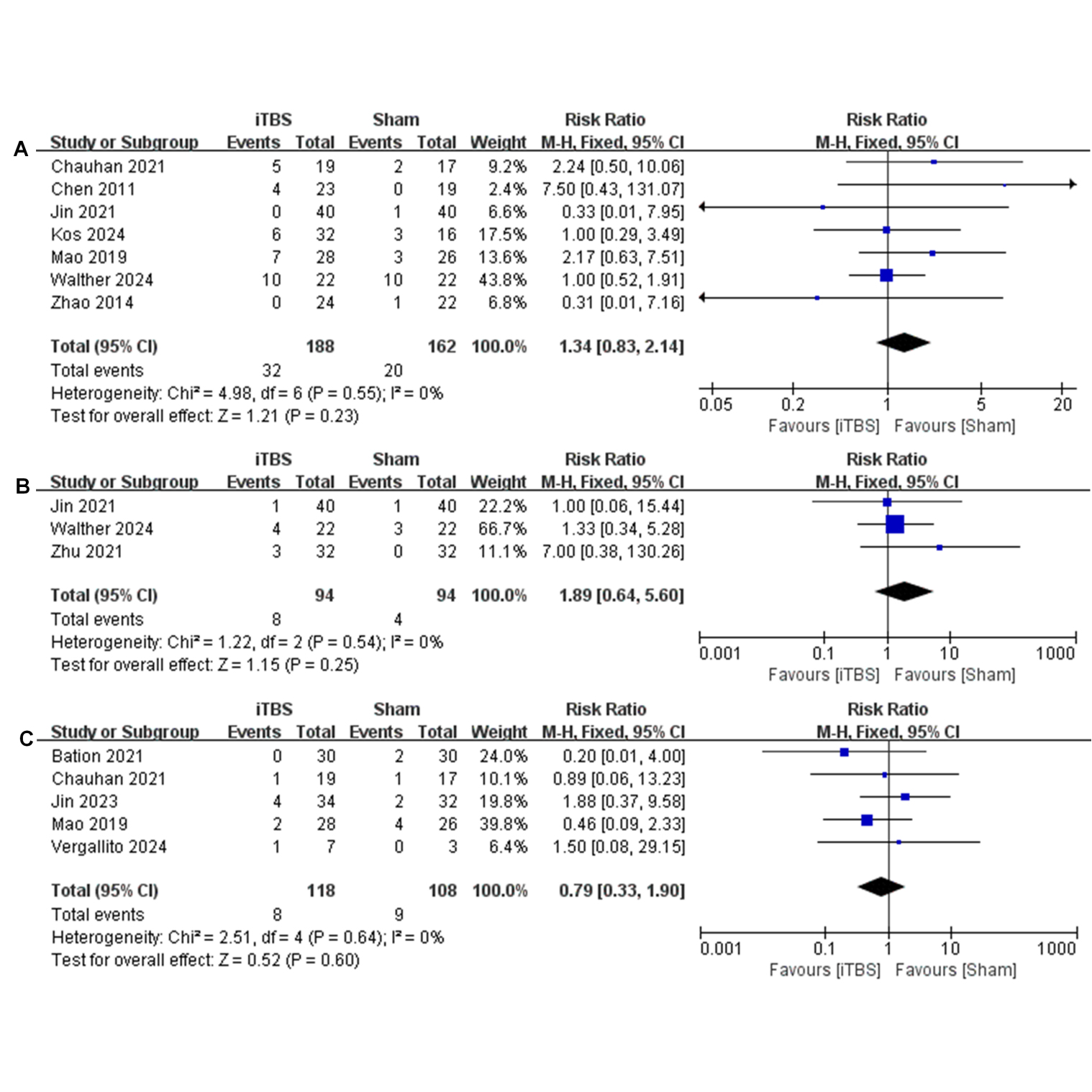
**Supplementary Figure 5: Forest plot of the risk of side effects associated with iTBS compared to sham control, stratified by the type of adverse event.** Data for each subgroup were pooled using a **Mantel-Haenszel fixed-effect model**. Each square represents the risk ratio (RR) for an individual randomized controlled trial; the horizontal line indicates its 95% confidence interval (CI), and the area of the square is proportional to the study’s weight in the meta-analysis. Pooled effect estimates are represented by diamonds with 95% CIs**. The sample sizes (iTBS/sham) for each subgroup are: (A) headache (N = 188/162), (B) dizziness (N = 94/94), and (C) other side effects (N = 118/108).** iTBS, intermittent theta burst stimulation; CI, confidence interval; RR, risk ratio; N, number of participants.


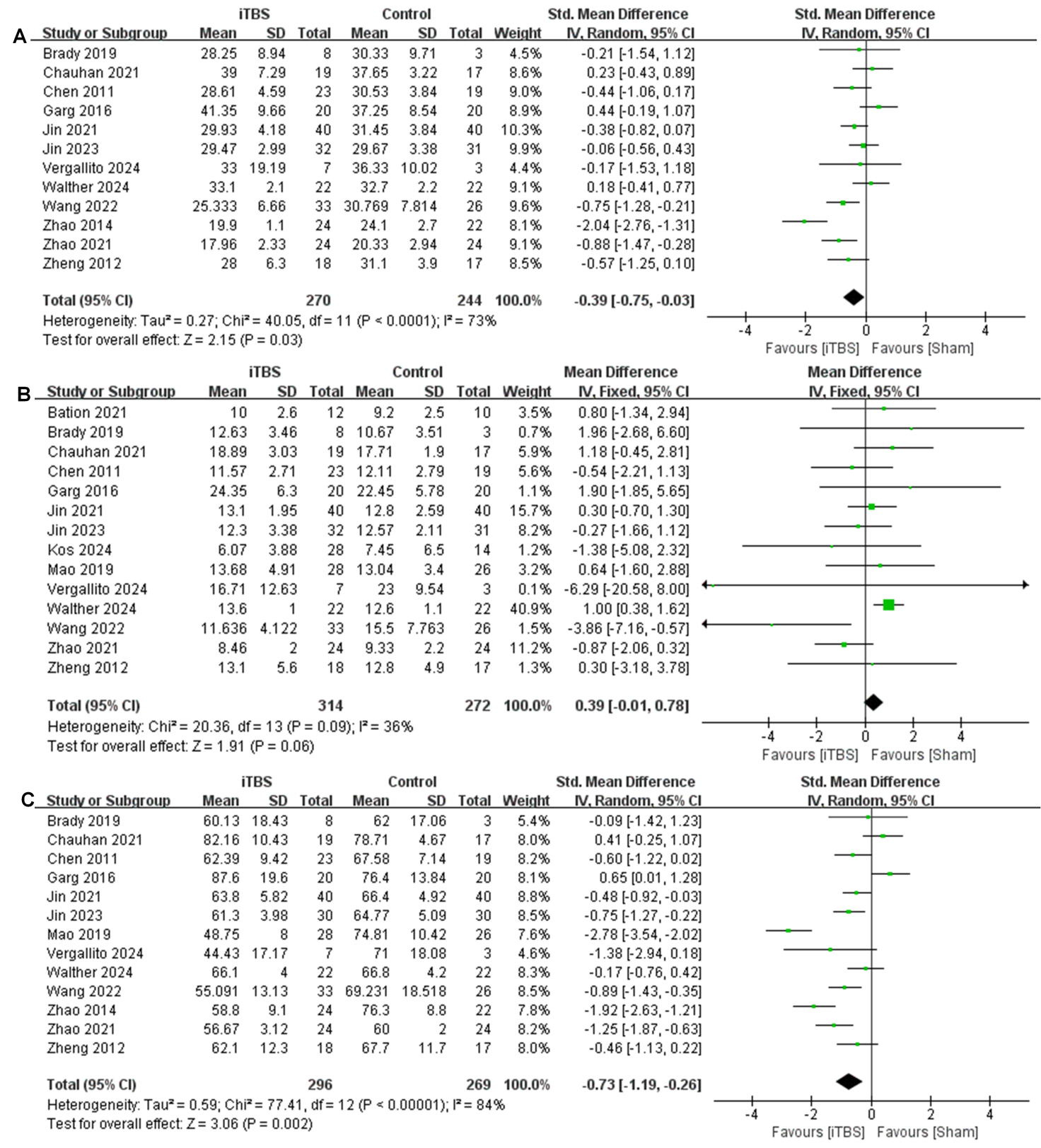


**Supplementary Figure 6: Forest plot of the effects of intermittent theta burst stimulation (iTBS) on Positive and Negative Syndrome Scale (PANSS) scores, stratified by symptom domain.** Data for each subgroup were pooled using inverse-variance methods with random- or fixed-effects models, as appropriate. Each square represents the effect size (MD or SMD, as appropriate) for an individual randomized controlled trial; the horizontal line indicates its 95% confidence interval (CI), and the area of the square is proportional to the study’s inverse-variance weight. Pooled subgroup estimates are represented by diamonds with 95% CIs. Heterogeneity was assessed using the *I*² statistic, and the overall effect was tested with a Z-test. **The sample sizes (iTBS/sham) for each subgroup are**: (A) general psychopathology score **(N = 270/244)**, (B) positive symptom score **(N = 314/272)**, and (C) total score **(N = 296/269)**. iTBS, intermittent theta burst stimulation; CI, confidence interval; MD, mean difference; SMD, standardized mean difference; N, number of participants; *I²*, heterogeneity statistic.

**
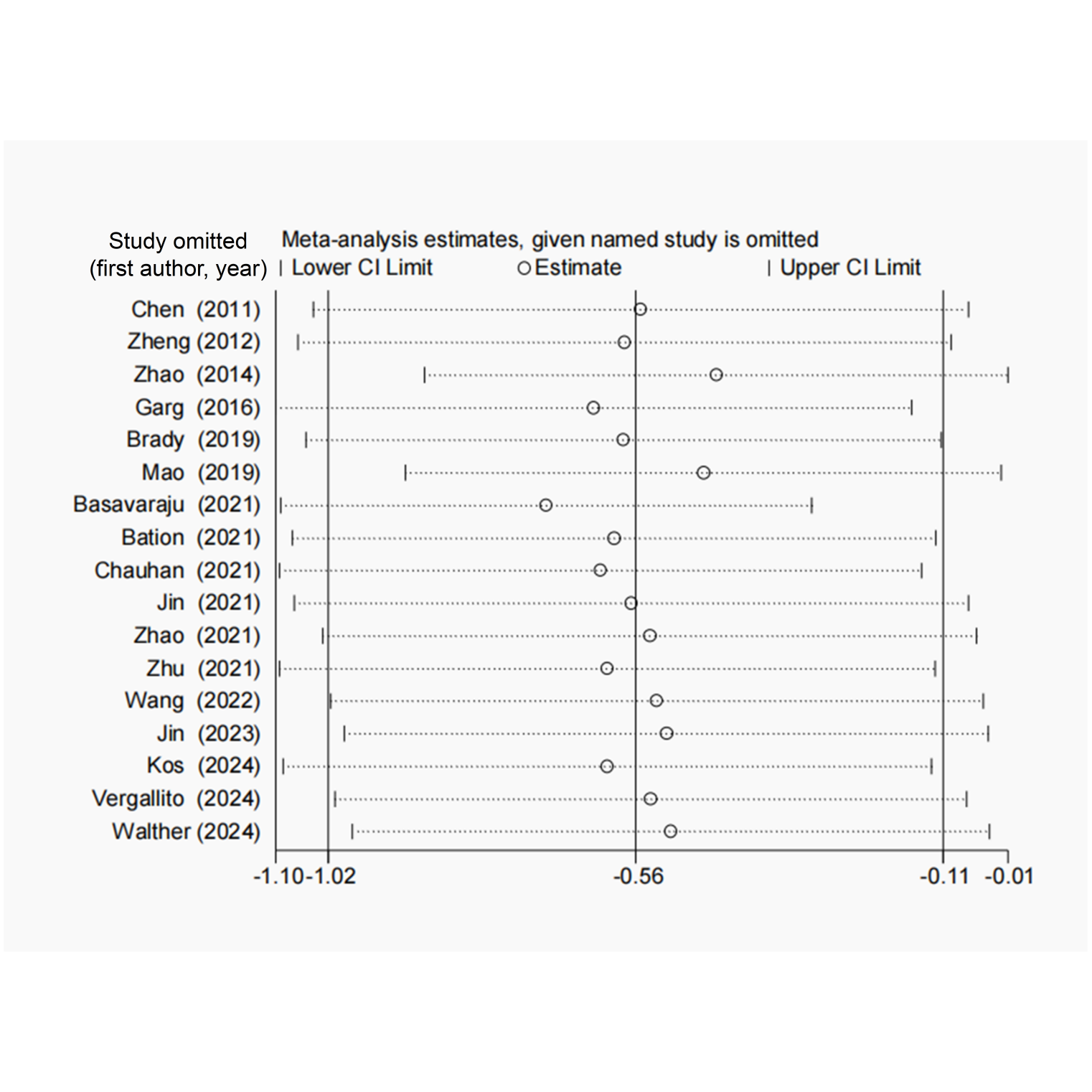
Supplementary Figure 7. Sensitivity analysis (leave-one-out analysis) of the pooled effect estimate.** The y-axis lists the 17 included studies by author name and publication year. Each data point (circle) represents the pooled standardized mean difference (SMD) from an inverse-variance weighted random-effects meta-analysis after omitting the corresponding study, and the horizontal lines indicate the associated 95% confidence intervals (CIs). Statistical significance of the pooled effect was assessed according to whether the 95% CI from the random-effects model excluded zero. The solid vertical line marks the overall pooled effect from the main analysis (SMD = −0.56, 95% CI −1.02 to −0.11). The leave-one-out analysis shows that removal of any single study does not materially change the magnitude, direction, or significance of the pooled effect, indicating that the overall conclusion is robust. Sample sizes: iTBS group (N = 405), sham group (N = 359). iTBS, intermittent theta burst stimulation; CI, confidence interval; SMD, standardized mean difference; N, number of participants.

**
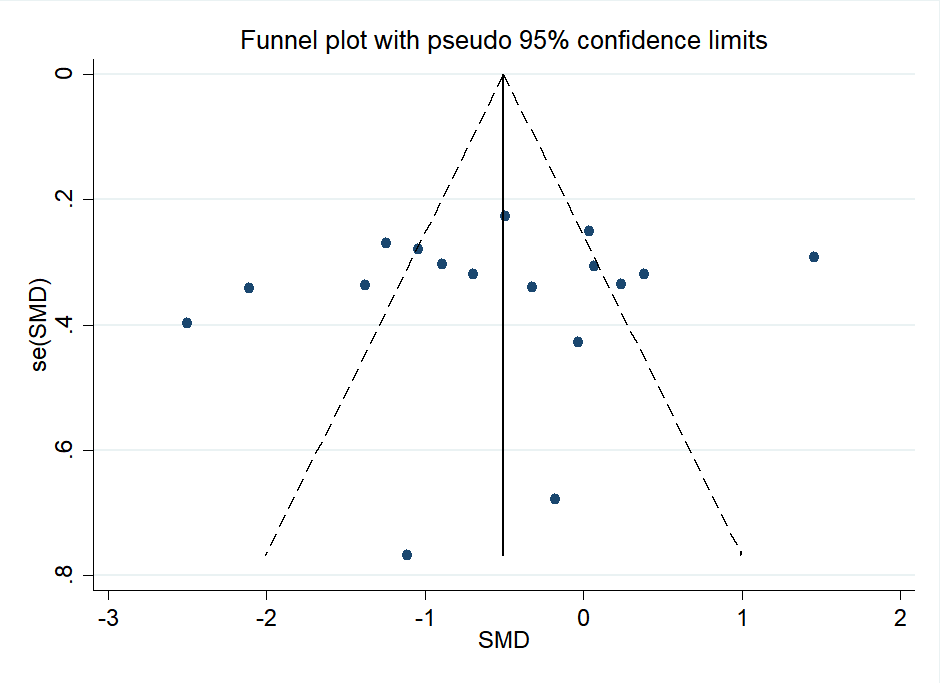
**

**Supplementary Figure 8: **Funnel plot assessing potential publication bias in the meta-analysis.** Each dot represents an individual randomized controlled trial, plotted as its standardized mean difference (SMD) against its standard error [se(SMD)]**.** Dashed lines indicate pseudo 95% confidence limits. The distribution of studies appears approximately symmetrical, and Egger’s regression test did not indicate significant small-study effects or substantial publication bias (*P* = 0.853). Sample sizes: iTBS group (N = 405), sham group (N = 359); total N = 764.** SMD, standardized mean difference; se(SMD), standard error of the standardized mean difference; iTBS, intermittent theta burst stimulation; N, number of participants.

**Supplementary Figure 9: Forest plot comparing antipsychotic medication usage between the iTBS and sham stimulation groups.** Data from individual randomized controlled trials were pooled using an inverse-variance weighted fixed-effect model. Each square represents the standardized mean difference (SMD) for an individual randomized controlled trial; the horizontal line indicates its 95% confidence interval (CI), and the area of the square is proportional to the study’s inverse-variance weight. The pooled effect estimate is represented by a diamond with its 95% CI. Heterogeneity was assessed using the *I*² statistic, and the overall effect was tested with a Z-test. The total sample sizes are N = 190 for the iTBS group and N = 175 for the sham group. iTBS, intermittent theta burst stimulation; CI, confidence interval; SMD, standardized mean
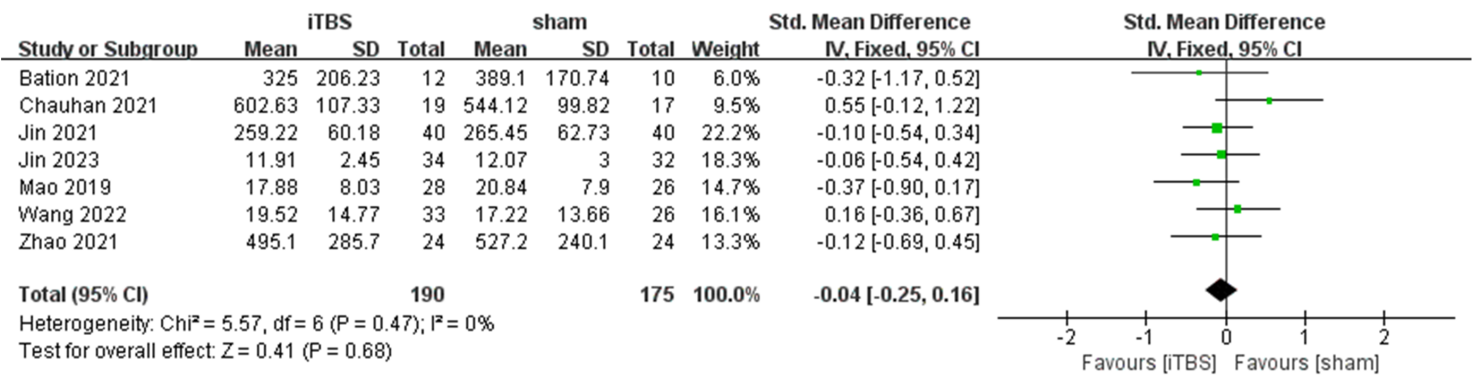
difference; N, number of participants; *I²*, heterogeneity statistic.

| **Supplementary Table 1. Study characteristics and patient sociodemographic factors.** | | | | | | | | | | | | | | | | | | | |
| --- | --- | --- | --- | --- | --- | --- | --- | --- | --- | --- | --- | --- | --- | --- | --- | --- | --- | --- | --- |
| **Author** | **Year** | **Study design** | **Baseline selection**  **Of Negative Symptoms** | **Diagnostic criteria** | **Participants**  **Comparison** | **Subject** | **mean age** | **age**  **SD** | **man** | **female** | **illness duration(year) mean** | **SD** | **Education**  **(Mean Year)** | **Education**  **(Year)SD** | **Country** | CPZ Eq mg（mean） | CPZ Eq mg（SD） | OPZ Eq mg（mean） | OPZ Eq mg（SD） |
| **Basavaraju** | **2021** | a double-blind, parallel, randomized, sham-controlled trial | schizophrenia | DSM-5 | Active | 30 | 31.17 | 9.90 | 24 | 6 | 8.43 | 5.60 | 11.47 | 3.70 | India | NA | NA |  |  |
|  |  |  | a score of three (moderate) or more on each of the five global rating items of SANS |  | Sham | 30 | 34.17 | 8.06 | 22 | 8 | 10.87 | 8.02 | 11.13 | 3.90 | India | NA | NA |  |  |
| **Bation** | **2021** | a randomized, double-blind study | schizophrenia with treatment-resistant negative symptoms | DSM-  IV-TR | Active | 12 | 42.33 | 9.44 | 12 | 0 | 15.00 | 5.86 | 11.45 | 2.54 | France | 325.00 | 206.23 |  |  |
|  |  |  | PANSS negative subscale ≥20 |  | Sham | 10 | 41.60 | 12.63 | 9 | 1 | 17.11 | 15.37 | 12.10 | 2.81 | France | 389.10 | 170.74 |  |  |
| **Brady** | **2019** | a randomized trial | schizophrenia or schizoaffective disorder | DSM-  IV-TR | Active | 8 | 35.55 | 10.50 | 8 | 3 | NA | NA | NA | NA | America | 614.20 | 606.50 |  |  |
|  |  |  |  |  | Sham | 3 | 35.55 | 10.50 | 8 | 3 | NA | NA | NA | NA | America | 614.20 | 606.50 |  |  |
| **Chauhan** | **2021** | a randomized rater blinded placebo control trial | Treatment-Resistant Schizophrenia | ICD-10 | Active | 19 | 41.74 | 8.80 | 7 | 12 | 16.05 | 5.50 | NA | NA | India | 602.63 | 107.33 |  |  |
|  |  |  |  |  | Sham | 17 | 39.35 | 8.20 | 8 | 9 | 13.00 | 6.90 | NA | NA | India | 544.12 | 99.82 |  |  |
| **Chen** | **2011** | a randomized, double-blind, sham-controlled study | schizophrenia | DSM-IV | Active | 23 | 37.40 | 11.80 | 16 | 7 | 15.21 | 13.43 | 12.00 | 2.20 | China | NA | NA |  |  |
|  |  |  | negative symptom factor score of PANSS≥20 |  | Sham | 19 | 39.70 | 13.30 | 11 | 8 | 13.36 | 13.62 | 11.00 | 2.60 | China | NA | NA |  |  |
| **Garg** | **2016** | a randomized rater blinded placebo control design | schizophrenia | ICD-10 | Active | 20 | 32.40 | 8.44 | 17 | 3 | 7.16 | 7.49 | NA | NA | India | NA | NA |  |  |
|  |  |  |  |  | Sham | 20 | 30.75 | 7.90 | 16 | 4 | 6.05 | 5.60 | NA | NA | India | NA | NA |  |  |
| **Jin** | **2021** | a randomized, double-blind study | schizophrenia | ICD-10 | Active | 40 | 48.65 | 9.65 | 16 | 24 | 8.90 | 4.02 | 7.20 | 2.48 | China | 259.22 | 60.18 |  |  |
|  |  |  |  |  | Sham | 40 | 47.75 | 10.57 | 19 | 21 | 8.40 | 4.31 | 6.40 | 2.39 | China | 265.45 | 62.73 |  |  |
| **Jin** | **2023** | a randomized controlled trial | schizophrenia | DSM-5 | Active | 34 | 47.60 | 9.46 | 18 | 16 | 8.83 | 3.95 | 7.53 | 2.36 | China |  |  | 11.91 | 2.45 |
|  |  |  |  |  | Sham | 32 | 47.47 | 10.99 | 19 | 13 | 8.40 | 4.29 | 7.00 | 2.30 | China |  |  | 12.07 | 3.00 |
| **Kos** | **2024** | a multi-center, randomized, placebo-controlled, and rater-blinded trial | schizophrenia or schizoaffective disorder | DSM-IV | Active | 32 | 33.45 | 8.34 | 24 | 8 | 8.07 | 5.43 | 17.10 | 2.90 | The Netherlands | NA | NA |  |  |
|  |  |  |  |  | Sham | 16 | 36.72 | 18.69 | 12 | 4 | 7.04 | 7.11 | 16.00 | 2.80 | The Netherlands | NA | NA |  |  |
| **Mao** | **2019** | a randomized controlled trial | schizophrenia | DSM-IV | Active | 28 | 52.75 | 7.00 | 19 | 9 | 27.68 | 9.20 | 9.11 | 1.20 | China |  |  | 17.88 | 8.03 |
|  |  |  | negative symptom factor score of PANSS≥18 |  | Sham | 26 | 53.50 | 5.50 | 15 | 11 | 27.54 | 9.92 | 9.27 | 1.80 | China |  |  | 20.84 | 7.9 |
| **Vergallito** | **2024** | a randomized controlled trial | schizophrenia | DSM-5 | Active | 6 | 40.33 | 11.20 | 4 | 2 | 11.83 | 11.51 | 13 | 0 | Italy | NA | NA |  |  |
|  |  |  |  |  | Sham | 3 | 32.30 | 8.30 | 2 | 1 | 4.67 | 0.58 | 12.33 | 1.15 | Italy | NA | NA |  |  |
| **Walther** | **2024** | 4-arm, double-blind, randomized, placebo-controlled clinical trial | schizophrenia spectrum disorders | DSM-5 | Active | 22 | 33.50 | 11.50 | 12 | 10 | 7.00 | 8.40 | 13.10 | 2.20 | Switzerland | NA | NA |  |  |
|  |  |  |  |  | Sham | 22 | 38.2 | 12.2 | 9 | 13 | 12.9 | 12.4 | 12.7 | 2.2 | Switzerland | NA | NA |  |  |
| **Wang** | **2022** | a randomized, pilot study | schizophrenia | DSM-  IV (SCID, Version 2.0) | Active | 33 | 23.79 | 5.33 | 15 | 18 | 4.56 | 3.36 | 12.09 | 2.79 | China |  |  | 19.52 | 14.77 |
|  |  |  |  |  | Sham | 26 | 24.15 | 4.62 | 11 | 15 | 4.62 | 3.79 | 11.92 | 2.19 | China |  |  | 17.22 | 13.66 |
| **Zhao** | **2014** | a double-blind randomized trial | schizophrenia | DSM-IV | Active  (iTBS) | 24 | 47.70 | 11.80 | 13 | 11 | NA | NA | 12.90 | 0.90 | China | NA | NA |  |  |
|  |  |  |  |  | Active  (10Hz) | 23 | 48.00 | 12.20 | 10 | 13 | NA | NA | 10.30 | 0.30 | China | NA | NA |  |  |
|  |  |  |  |  | Active  (20Hz) | 24 | 49.10 | 10.60 | 11 | 13 | NA | NA | 12.50 | 0.30 | China | NA | NA |  |  |
|  |  |  |  |  | Sham | 22 | 46.70 | 13.10 | 12 | 10 | NA | NA | 13.80 | 0.10 | China | NA | NA |  |  |
| **Zhao** | **2021** | a double-blind randomized trial | schizophrenia | ICD-10 | Active | 24 | 62.50 | 3.32 | 15 | 9 | 31.33 | 8.87 | 9.25 | 2.33 | China | 495.10 | 285.70 |  |  |
|  |  |  |  |  | Sham | 24 | 64.04 | 3.61 | 17 | 7 | 35.00 | 8.85 | 9.50 | 2.39 | China | 527.20 | 240.10 |  |  |
| **Zheng** | **2012** | a randomized, double-blind study | schizophrenia | CCMD-3 | Active  (iTBS) | 18 | 56.40 | 9.30 | 18 | 0 | 32.90 | 8.10 | 2.20 | 0.70 | China | NA | NA |  |  |
|  |  |  |  |  | Active  (10Hz) | 19 | 56.50 | 7.40 | 19 | 0 | 33.20 | 10.00 | 2.40 | 0.60 | China | NA | NA |  |  |
|  |  |  |  |  | Active  (20Hz) | 19 | 56.80 | 5.40 | 19 | 0 | 33.60 | 6.50 | 2.40 | 0.80 | China | NA | NA |  |  |
|  |  |  |  |  | Sham | 17 | 55.60 | 5.80 | 17 | 0 | 31.70 | 7.20 | 2.40 | 0.60 | China | NA | NA |  |  |
| **Zhu** | **2021** | a multicenter, randomized, sham-controlled, double-blinded trial | schizophrenia | ICD-10 | Active | 32 | 35.16 | 7.14 | 18 | 14 | 15.40 | 7.80 | NA | NA | China | NA | NA |  |  |
|  |  |  |  |  | Sham | 32 | 35.34 | 6.12 | 14 | 18 | 15.84 | 6.47 | NA | NA | China | NA | NA |  |  |
| Abbreviations: CPZ Eq, Chlorpromazine Equivalent; CCMD, Chinese Classification of Mental Disorders; DSM, Diagnostic and Statistical Manual of Mental Disorders; Hz, hertz; ICD, International Classification of Diseases; iTBS, intermittent theta burst stimulation; OPZ Eq, Olanzapine Equivalent; NA, not available. | | | | | | | | | | | | | | | | | | | |

| **Supplementary Table 2. Parameters of transcranial magnetic stimulation included in the study.** | | | | | | | | | | | |
| --- | --- | --- | --- | --- | --- | --- | --- | --- | --- | --- | --- |
| **Author** | **Year** | **Machine origin** | **Coil** | **Navigation pattern** | **Target** | **Intensity (MT%)** | **Pulses Per Session** | **Sessions per day (working days)** | **Total Number of sessions** | **Duration (week)** | **Total Number of pulses** |
| **Basavaraju** | **2021** | MagPro X100 (MagVenture, Farum, Denmark) | figure-of-8 Cool-B65 AP MagVenture coil | MRI-Navigated | cerebellum（vermal part） | 100% | 600 | 2 | 10 | 1 | 6000 |
| **Bation** | **2021** | Magpro X100 (Magventure, Mag2Health, France). | figure-8 coil | 6 cm anterior to the scalp position corresponding to the motor cortex | L-DLPFC | 80% | 990 | 2 | 20 | 2 | 19800 |
| **Brady** | **2019** | Magpro X100  (Cool B65 A/P, Magventure, Denmark) | figure-8 coil | Brainsight frameless stereotaxic system | cerebellum（vermal part） | 100% | 600 | 2 | 10 | 1 | 6000 |
| **Chauhan** | **2021** | MagPro-R30 (MagVenture) | figure-of-8-shaped coil | 10–20 EEG system | cerebellum（vermal part） | 80% | 600 | 2 | 10 | 1 | 6000 |
| **Chen** | **2011** | Magpro X100 (Denmark) | the circular magnet version | NA | L-DLPFC | 80% | 2400 | 1 | 20 | 4 | 48000 |
| **Garg** | **2016** | Magstim Rapids device | double-cone coils | 1 cm below the ‘inion’ | cerebellum（vermal part） | 100% | 600 | 1 | 10 | 2 | 6000 |
| **Jin** | **2021** | RT-100 | the circular magnet version | NA | L-DLPFC | 100% | NA | NA | NA | NA | NA |
| **Jin** | **2023** | M-100 (Brain Ultimate, Atlanta, USA) | 70-mm air-cooled butterfly coil | TMS Navigation System | L-DLPFC | 100% | 1800 | 1 | 20 | 4 | 36000 |
| **Kos** | **2024** | Medtronic MagPro machines (one X100 and two R30 machines) | figure-of-eight | 10–20 EEG system | R-DLPFC | 80% | 990 | 1 | 10 | 2 | 9900 |
| **Mao** | **2019** | MagPro R100 (Denmark) | NA | NA | L-DLPFC | 80% | NA | 1 | 20 | 4 | NA |
| **Vergallito** | **2024** | MagStim Rapid2 stimulator | figure-of-eight coil | 10–20 EEG system | L-DLPFC | 100% | 600 | 1 | 15 | 3 | 9000 |
| **Walther** | **2024** | MagPro X100 | MCF-B70 coil | NA | Bilateral supplementary motor area | 80% | 1200 | 1 | 15 | 3 | 18000 |
| **Wang** | **2022** | MagStim Rapid2 stimulator | figure-eight coil | MRI tracking and navigation system | L-DLPFC | 80% | 600 | 3 | 10 | 2 | 36000 |
| **Zhao** | **2014** | MagPro R100 | MF-125 round coil | NA | L-DLPFC | 80% | 2400 | 1 | 20 | 4 | 48000 |
| **Zhao** | **2021** | NA | NA | NA | L-DLPFC | 100% | 600 | 1 | 20 | 4 | 12000 |
| **Zheng** | **2012** | Magpro X100 | MF-127 round coil | NA | L-DLPFC | 80% | 1200 | 1 | 5 | 1 | 6000 |
| **Zhu** | **2021** | MagPro X100 (Medtronic Dantec NeuroMuscular, Skovlunde, Denmark) | figure-eight coil | 1 cm below the ‘inion’ | cerebellum（vermal part） | 100% | 600 | 1 | 10 | 2 | 6000 |
| Abbreviations: EEG, electroencephalogram; L-DLPFC, left dorsolateral prefrontal cortex; MT, motor threshold; NA, Not Available; R-DLPFC, right dorsolateral prefrontal cortex. | | | | | | | | | | | |

| **Supplementary Table 3. Safety and side effects of iTBS treatment for patients with schizophrenia** | | | | | |
| --- | --- | --- | --- | --- | --- |
|  |  | **Side effects during treatment phase** | | **Side effects during follow-up period** | |
| **Author** | **Year** | **iTBS** | **Sham** | **iTBS** | **Sham** |
| Basavaraju | 2021 | 2mania/hypomania;  1 neck pain/  discomfort | NO | NO | NO |
| Bation | 2021 | mild headache | mild headache | NO | 2 displayed an exacerbation of positive symptoms |
| Brady | 2019 | NO | NO | NO | NO |
| Chauhan | 2021 | 5 mild headache | 2 mild headache | 1 overnight  aggression after 2 sessions | 1 lack of benefit after 3 sessions |
| Chen | 2011 | 4 headaches | NO | NA | NA |
| Garg | 2016 | NO | NO | NO | NO |
| Jin | 2021 | 1 dizziness, 1 stabbing pain, 2 scalp numbness | 1 headache,  1 dizziness,  1 scalp numbness | NA | NA |
| Jin | 2023 | 10 numb-headedness | 3 numb-  headedness | 4 | 2 |
| Kos | 2024 | 6 mild headache and muscle contraction. | 3 headache | NO | NO |
| Mao | 2019 | 7 headache | 3 headache | 2 | 4 |
| Vergallito | 2024 | NO | NO | 1 | NO |
| Walther | 2024 | 4 dizziness | 3 dizziness | NA | NA |
|  |  | 10 headache or neck pain | 10 headache or neck pain | NA | NA |
|  |  | 5 Fatigue | 4 Fatigue | NA | NA |
| Wang | 2022 | NA | NA | NA | NA |
| Zhao | 2014 | 2 insomnia | 1 severe  headache | NA | NA |
| Zhao | 2021 | 1 cannot  tolerate  treatment | NO | NA | NA |
| Zheng | 2012 | NO | NO | NA | NA |
| Zhu | 2021 | 3mild dizziness, pain, nausea | NO | NO | NO |
| Abbreviations: iTBS, intermittent theta burst stimulation; NA, not available. | | | | | |
